# Motion Recognition Based on Deep Learning and Human Joint Points

**DOI:** 10.1155/2022/1826951

**Published:** 2022-05-10

**Authors:** Junping Wang

**Affiliations:** Foundation Department, Huaibei Vocational and Technical College, Huaibei 23500, China

## Abstract

In order to solve the problem that the traditional feature extraction methods rely on manual design, the research method is changed from the traditional method to the deep learning method based on convolutional neural networks. The experimental results show that the larger average DTW occurs near the 55th calculation, that is, about the 275th frame of the video. In the 55th calculation, the joint angle with the largest DTW distance is the right knee joint. A multiscene action similarity analysis algorithm based on human joint points has been realized. In the fitness scene, by analyzing the joint angle through cosine similarity, the time of fitness key posture in the action sequence can be recognized. In the sports scene, through the similarity analysis of joint angle sequences by the DTW algorithm, we can get the similarity between people's actions in the sports video and the joint positions with large differences in some time intervals, and the real validity of the experiment is verified. The accuracy of motion recognition before and after the improvement is 95.2% and 97.1%, which is 0.19% higher than that before the improvement. The methods and results are widely used in the fields of sports recognition, movement specification, sports training, health management, and so on.

## 1. Introduction

**Figure 1:**
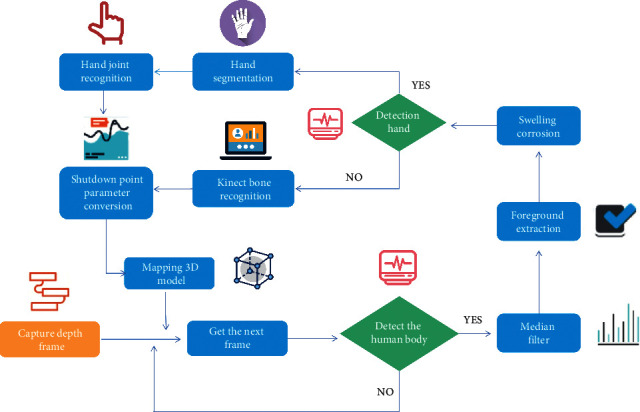
Motion recognition of human joint points.

With the development of deep learning and the mobile Internet, more and more deep learning applications begin to be deployed to mobile devices to provide services for people. In these applications, the analysis of people's posture and actions in the image is the focus of human-computer interaction, so human joint point detection, as a key technology, has become a research hotspot. In the current human joint point detection methods, more complex network structures are often designed because of the emphasis on improving the accuracy of the model. Therefore, there are usually problems of large model calculation times and large space occupations. However, due to the limited computing power and storage of mobile devices, such models are difficult to deploy to devices. Aiming at the problem of human joint point detection and aiming at the final deployment of the model to the mobile terminal equipment, it is committed to studying the human joint point detection model that can balance the calculation consumption and model accuracy and can be deployed and used in the mobile terminal, and on the basis of that, it realizes the function of the human action similarity analysis algorithm in various scenes. Tobias et al. studied and analyzed the classic pedestrian detection method. Based on fast R-CNN (fast region-based revolutionary neural network), they improved it from three aspects affecting the pedestrian detection model, used a more scientific residual network bottleneck structure in the network structure, and introduced extended convolution to increase the receptive field [1]. Gong and Shu proposed the introduction of mixup, which has a good effect in the field of image classification, to expand the data and improve the generalization performance of the model. In terms of the loss function, aggregation loss is added to the original loss function to improve the dense occlusion. Achieve high pedestrian detection accuracy on the coco2017 dataset [2]. For the human joint point detection network used in this study, Wang et al. improved the processing method of the loss function to solve the difficult human joint points. Based on the current research, different processing methods are used for the output of different level networks. The first level network uses ordinary L2 loss to process all human joint points, and the second level network uses a batch level difficult joint point mining method to process difficult human joint points, as shown in Figure 1 [3].

## 2. Methods

### 2.1. Faster R-CNN Algorithm

**Figure 2:**
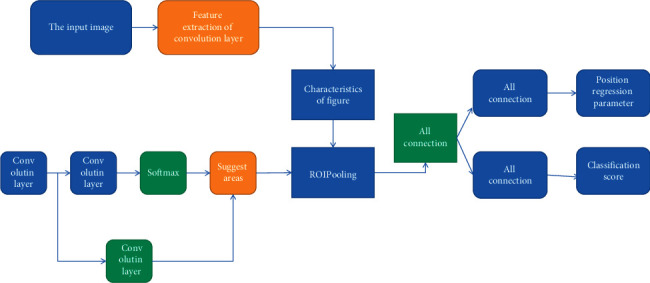
Schematic diagram of faster-R-CNN frame.

The overall framework of faster R-CNN is shown in Figure 2. In fact, the framework adds an RPN network structure to the faster R-CNN network to generate regions of interest. The fast R-CNN part extracts the feature map that can represent the key information of the image through multiple convolution layers, and the RPN network is used to generate the region of interest. The generated region of interest and the extracted feature map are transmitted to the ROI pooling layer as input, and the feature map of the region of interest with the uniform size is output. Finally, the feature map of the region of interest is classified through the full connection layer. At the same time, the position of the target frame is corrected by target frame position regression, and finally, accurate target detection is realized [4–6]. Several core components of the algorithm framework will be briefly described below.

**Figure 3:**
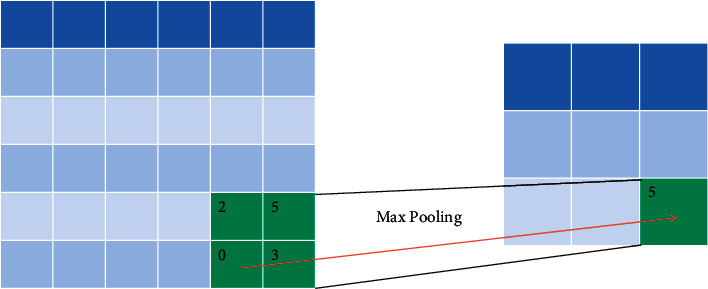
Schematic diagram of ROI pool layer process.

The region of interest extracted by the RPN network corresponds to Figure 2 and 3 × 3 convolution up to the recommended part of the area. Regions of interest refer to the proposed regions considered to have targets in the process of target detection, and then, the detection network will carry out further target detection and target frame position regression for these regions. In the traditional region-of-interest generation methods, selective search algorithm is often used. The algorithm takes the similarity between pixels on the image as the standard to construct the minimum spanning tree between pixels so that the pixels with high similarity can be merged into the same region, which is considered as the region with the same semantics, that is, the region of interest of the target. Although this kind of traditional method has a simple structure, there are a lot of repeated calculations, which reduces the speed of the whole detection algorithm, and this kind of algorithm cannot effectively extract the deeper features of the picture [3, 7, 8]. In order to solve the problems of the above traditional methods, the RPN network is introduced to generate the target region of interest. Its network structure is shown in Figure 3. The main purpose of the ROI pooling layer is to unify the feature map size of the region of interest because the region of interest is selected by the RPN network from many anchor candidate boxes. Therefore, the size of the region-of-interest frame output by the RPN network is not uniform, so the size of its corresponding feature map is not uniform. However, the subsequent fully connected networks need a unified size input, so the feature map size corresponding to these regions of interest is scaled to a unified size through the ROI pooling layer.

The general operation steps of the ROI pool layer are as follows:

1. According to the position of the input region of interest in the original map, the corresponding feature map is mapped
2. Divide the mapped area into equal nxm units according to the input size (nxm) required by the subsequent full connection layer
3. Max pooling is performed on the eigenvalues in each cell, that is, the maximum eigenvalue of the cell is calculated as the eigenvalue of the cell after pooling

ROI pooling layer not only skillfully unifies regions of interest with different sizes but also further reduces the dimension of features, refines core features, and greatly improves the processing speed of the network.

In the previous section, the RPN region of the interest generation process uses nine sizes of anchor candidate boxes by default. These nine sizes are set to enhance the recognition generalization ability of the network for various sizes and classes of objects in the image. In this study, only fast R-CNN is used to identify the specific category of “people,” so the size of the candidate box can be adjusted. According to the common pedestrian sizes in the INRIA dataset, the 9 candidate box sizes' sets are shown in Table 1, and their proportions are close to 0.4, which is close to the aspect ratio of normal upright pedestrians [9–11].

The classified network part of faster R-CNN includes two branch networks. One of them is used to calculate the confidence score of the target in the target box (character detection is a binary classification problem, so the output dimension is 2); the other branch is used to calculate the regression parameters of the region of interest box, which are used to correct the position coordinates of the region of interest so as to make the region of interest closer to the real target position. The calculation formula for correcting the region of interest by using the regression parameters is as follows:

(1) x=wa×tx+xa,y=ha×ty+ya,h=ha×  expth,w=w×aexptw,

where *x*_*a*_, *y*_*a*_, *w*_*a*_, and *h*_*a*_, respectively, represent the horizontal and vertical coordinates and width and height of the center point of the regression frame coordinates, *x*, *y*, *w*, and *h*, respectively, represent the horizontal and vertical coordinates, width, and height of the center point of the prediction frame coordinate, and *x*^*∗*^, *y*^*∗*^, *w*^*∗*^, and *h*^*∗*^, respectively, represent the horizontal and vertical coordinates and width and height of the center point of the GT coordinate. Equation (1) shows that the correction calculation is carried out from the position coordinates of the region of interest *A* = (*x*_*a*_, *y*_*a*_, *w*_*a*_, *h*_*a*_). Before correction through a set of regression parameters *t*_*x*_, *t*_*y*_, *t*_*w*_, and *t*_*h*_ to obtain the corrected regression window *g* = (*x*, *y*, *W*, *H*) so that the regression window is closer to the marked real box, GT = (*x*^*∗*^, *y*^*∗*^, *w*^*∗*^, *h*^*∗*^). Therefore, the purpose of calculating the offset branch network is to predict the four regression parameters *t*_*x*_, *t*_*y*_, *t*_*w*_, and *t*_*h*_.

When the training network predicts the four parameters *t*_*x*_, *t*_*y*_, *t*_*w*_, and *t*_*h*_, it is necessary to input the real values corresponding to the four groups of parameters *t*_*x*_^*∗*^, *t*_*y*_^*∗*^, *t*_*w*_^*∗*^, and *t*_*h*_^*∗*^ to the training model [12–14]. This group of real values needs to be calculated together with the regression box position *G* = (*x*, *y*, *w*, *h*) output by the network and the real target box position GT = (*x*^*∗*^, *y*^*∗*^, *w*^*∗*^, *h*^*∗*^) marked by the training set data. The calculation method is as follows:

(2) $$\begin{array}{c} \left\{\begin{array}{c} x^{*}=w_{a}\times t_{x}^{*}+x_{a},\\ y^{*}=h_{a}\times t_{y}^{*}+y_{a},\\ w^{*}=w_{a}\times \;\exp\left(t_{w}^{*}\right),\\ h^{*}=h_{a}\times \;\exp\left(t_{h}^{*}\right). \end{array}\right. \end{array}$$

In the whole training process of faster R-CNN, fast R-CNN and RPN are trained separately, so it is necessary to design loss functions, respectively. When designing the loss function, the recognition accuracy of the target object and the proximity between the target frame position and the real frame position are mainly considered. Therefore, the loss function of RPN is defined as follows:

(3) $$\begin{array}{c} L\left(\left\{p_{i}\right\},\left\{t_{i}\right\}\right)=\frac{1}{N_{\text{cls}}}\sum_{i}l_{\text{cls}}\left(p_{i},p_{i}^{*}\right)+\lambda \frac{1}{N_{\text{reg}}}\sum_{i}L_{\text{reg}}\left(t_{i},t_{i}^{*}\right),\\ \text{smooth}\left(x\right)=\left\{\begin{array}{c} 0.5^{x2}\times \frac{1}{\sigma^{2}}\text{if}\left|x\right|<\frac{1}{\sigma^{2}},\\ \left|x\right|-0.5\text{else,} \end{array}\right. \end{array}$$

where *p*_*i*_ represents the predicted classification probability of the *i*th target box. When the *i*th target box is a positive sample, *p*_*i*_^*∗*^, otherwise *C* = 0. In the training process, the way to judge whether it is a positive sample or a negative sample is as follows: when the intersection and union ratio between the target frame and GT (group true, real label frame) is greater than 0.7, it is considered that the target frame is a positive sample frame, and if the intersection and union ratio is less than 0.3, it is considered that it is a negative sample frame, i.e., the target frame that does not belong to the positive sample and does not belong to the negative sample does not participate in the training. *t*_*i*_ represents the regression parameters of the target box, and *t*_*i*_^*∗*^ represents the regression parameters of GT [15]. The conversion formula between the horizontal and vertical coordinates, the width and height of the center point corresponding to the regression parameters, and the frame position are shown in equation (1). *N*_cls_ represents the number of training sets used for a batch gradient descent, *N*_reg_ represents the number of target box regression parameters generated by a batch gradient descent. *N*_cls_, *N*_reg_, and *λ* are used for normalization, and *λ* is the balance factor to prevent the gap between *N*_cls_ and *N*_reg_ from being too large, generally *λ* ∼ 10. *L*_reg_ and *L*_cls_ are calculated as follows:

(4) $$\begin{array}{c} L_{\text{cls}}\left(p_{i},p_{i}^{*}\right)=\log\left[p_{i}^{*}p_{i}+\left(1-p_{i}^{*}\right)\left(1-p_{i}\right)\right], \end{array}$$

(5) $$\begin{array}{c} L_{\text{reg}}\left(t_{i},t_{i}^{*}\right)=\sum_{i\in \left\{x,y,w,h\right\}}{\text{smooth}}_{Li}\left(t_{i}-t_{i}^{*}\right). \end{array}$$

Among them, formula (4) is the loss function of target classification, which uses the classical binary logarithmic loss function; equation (5) is a loss function for the regression of the target frame position. This function uses the Smooth Li loss function, and its calculation formula is as follows:

(6) $$\begin{array}{c} {\text{smooth}}_{Li}\left(x\right)=\left\{\begin{array}{c} 0.5^{x^{2}}\times \frac{1}{\sigma^{2}}\text{if}\left|x\right|<\frac{1}{\sigma^{2}},\\ \left|x\right|-0.5\mathrm{else}\text{.} \end{array}\right. \end{array}$$

Among them, the function controls the smooth area through a parameter *σ*. Generally, *σ* = 3 when training RPN and *σ* = 1 when training fast R-CNN. Fast R-CNN itself is suitable for multicategory target classification, so the original Fast R-CNN module uses a multicategory cross-line loss function at the classification loss function. In this subject, fast R-CNN is introduced into the character target detection task, so it is a binary classification problem. Formula (4) can still be used as the classification loss function [16–18]. The position regression loss function of fast R-CNN is also consistent with the above RPN. Therefore, for this model, fast R-CNN and RPN use exactly the same loss function, as shown in formula (3).

The general idea of K-means algorithm is to select *C* samples from the training set as the center of the cluster, and calculate the distance between all sample points and the center of the *C* cluster. For each sample point, it is divided into the cluster to which the cluster center with the smallest distance value belongs. After such a clustering, the cluster center is recalculated and updated for each cluster. In this way, it continues to iterate until the model reaches satisfactory convergence accuracy. The algorithm flow is as follows:

(1) Set the initial value for each cluster center according to the set number of clusters, and generally, randomly select a group of samples as the initial cluster center *μ*_1_, *μ*_2_ … *μ*_*c*_.
(2) Update the label of the cluster to which all samples belong. The cluster label of the *i*th sample is *y_i_*. In this study, two feature components are set for the location of the annotation target box *x*_*i*_=(*x*_*i*_^1^, *x*_*i*_^2^). The first dimension is the area size of the dimension box, and the second dimension is the width height ratio of the dimension box. By calculating the Euclidean distance between the eigenvectors composed of these two values, we can better represent the similarity of the two-position dimension boxes in size and size. The calculation formula is as follows:
  (7) $$\begin{array}{c} y_{i}=\text{argmin dist}\left(x_{i},\mu_{j}\right)j\in \left[1,c\right],\\ \text{dist}\left(x_{i},\mu_{j}\right)=\sqrt{\sum_{k=1}^{2}{\left|x_{i}^{k}-u_{j}^{k}\right|}^{2}}. \end{array}$$
(3) Update the center point of each cluster. The formula is as follows:
  (8) $$\begin{array}{c} \mu_{j}=\frac{\sum_{k\in s_{j}}x_{k}}{k_{j}},j=1,2\ldots c, \end{array}$$
where *k*_*j*_ represents the number of samples assigned to the *j*th cluster in this round of iteration, *s*_*j*_ represents the set of sample points assigned to the *j*th cluster, and *k*_*j*_ represents the number of samples in set *s*_*j*_.
(4) Repeat steps 1 to 3 until the classification ability of the cluster center makes the model achieve convergence accuracy.

Through the above K-means clustering training process, taking the size and height-width ratio of the annotation box in the human keypoint detection dataset as the two dimensions of the feature vector, as the cluster classification standard, the classification results are obtained. For each cluster in the classification results, the average dimension of the annotation box in the same cluster is obtained, so as to obtain a group of anchor candidate box dimensions representative of the person size in the human keypoint dataset. In this study, the character annotation box provided by the MS COCO dataset is clustered [19–22]. Considering that the characters have rich actions, resulting in a large difference in the vertical and horizontal proportion of characters, the number of cluster clusters is increased to 13, and the dimension of the dimension box in each cluster is averaged. Finally, the candidate dimension of the anchor point is obtained, as shown in Table 2.

In order to solve the defects of the traditional nonmaximum suppression algorithm, the nonmaximum suppression algorithm has been improved. The confidence of the multitarget scene is appropriately reduced, and a variety of calculation methods are introduced to adjust the punishment methods of the confidence. Finally, experiments are conducted to analyze which punishment function calculation method can better improve the recognition performance of the algorithm in dense character scenes and reduce the sensitivity of the model to the confidence threshold. Three kinds of common weight calculation methods are selected as the discussion objects of this study, which are the linear function, the Gaussian function, and the exponential function. In this study, a penalty value is calculated through these three kinds of functions. When a candidate box intersects with the marked box and larger than the threshold appears in the process of nonmaximum suppression, its confidence is punished, so as to further judge whether to retain the candidate box, where *f* represents the type of penalty function used, *N* is the intersection union ratio threshold, and threshold represents the confidence threshold, which is used to judge whether the confidence after punishment meets the conditions that need to be eliminated. Next, the penalty function is defined according to three types of weighting methods.

The first is the linear penalty function, which is defined as follows:

(9) $$\begin{array}{c} s_{i}=\left\{\begin{array}{cc} s_{i}, & \text{Iou}\left(b_{m},b_{i}\right)<N_{1},\\ a.s_{i}\left(1-\text{IOU}\left(b_{m},b_{i}\right)\right), & \text{IOU}\left(b_{m},b_{i}\right)>N_{1}, \end{array}\right. \end{array}$$

where *a* is a coefficient weight, ranging from 0 to 1, used to control the intensity of punishment, *s* represents the confidence score of the candidate frame to be judged, *b*_*m*_ represents the position coordinate of the candidate frame with the highest confidence score discharged in this round of iteration, and *b*_*i*_ represents the position coordinate of the candidate frame currently being judged. When the intersection ratio of the two is greater than the threshold *N*_1_, it is necessary to punish the confidence of the candidate box. Different values of a and *N*_1_ will lead to different degrees of punishment. The sensitivity of parameters will be analyzed in subsequent experiments.

The second is the Gaussian penalty function, which is modified to wait for the penalty function:

(10) $$\begin{array}{c} s_{i}=s_{i}e^{-\text{Iou}{\left(b_{m},b_{i}\right)}^{2}/\sigma }, \end{array}$$

where *σ* represents the punishment for confidence, *s*_*i*_ represents the confidence score of the candidate box currently being judged, *b*_*m*_ represents the position coordinate of the candidate box with the highest confidence score discharged in this round of iteration, and *b*_*i*_ represents the position coordinate of the candidate box currently being judged.

The third is the exponential penalty function, which has a smoother transition at the threshold point than the linear function and can maintain a higher confidence than the Gaussian function at the stage of relatively low intersection and union. Its calculation formula is as follows:

(11) $$\begin{array}{c} s_{i}=\left\{\begin{array}{cc} s_{i}, & \text{IOU}\left(b_{m},b_{i}\right)<N_{1},\\ s_{i}e^{\left(N_{I-\text{IOU}\left(b_{m},b_{i}\right)}\right),} & \text{IOU}\left(b_{m},b_{i}\right)>N_{1}. \end{array}\right. \end{array}$$

Bring the above three penalty functions into the whole nonmaximum suppression process (*F* function in formula (10)). Each time the intersection and union ratio is judged, the above three types of penalty functions are introduced to calculate the confidence after punishment. If the confidence is still greater than the set confidence threshold, the candidate box is retained as the region of interest; otherwise, the candidate box is eliminated.

### 2.2. Deployment and Application of the Human Key Point Detection Model

In neural networks, quantization technology is usually used to reduce the accurate representation of weight and selectively reduce the stored and calculated activation value, so as to reduce the size of the model and improve the reasoning efficiency of the model. The simplest quantization strategy is to reduce the weight to 8 bits during reasoning. At present, in the field of deep learning, the data type used is the 32 bit (bit) float type, and the bit bits are directly related to the data range they can carry and express, as shown in Table 3.

There is no doubt that using a lower digit data type will reduce the representation range and cause a certain loss of accuracy after quantization. At present, the commonly used computing chips often design and adopt more special floating-point computing cores, while the number of floating-point computing cores on the chips of mobile devices and embedded devices is limited, so the computing power of floating-point data is often the bottleneck of computing consumption. The advantage of the low-bit data type generally lies in the speed of calculation, and the memory consumption can be directly reduced. For 8 bits quantization, the actual value and the quantized value are operated by mapping. The general mapping relationship is as follows:

(12) $$\begin{array}{c} r=\frac{T\text{ max}-T\text{ min}}{\left(2^{B}-1\right)-0}\times \left(q-z\right)\\ =S\times \left(q-z\right), \end{array}$$

where *r* is the actual value (generally of float32 type), *q* is its quantized representation (integer data of *b* bits, such as uint8 and uint32), *s* (float32) is used to represent the scaling factor, and *Z* (uint) is the factor used to adjust the offset.

**Figure 4:**
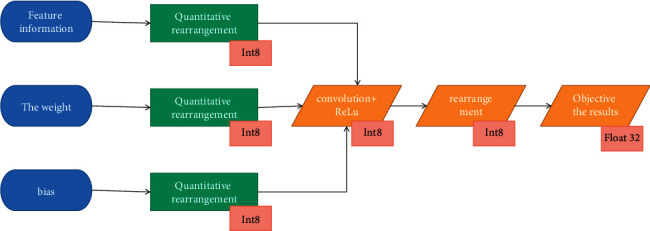
Quantitative model inference process.

In the frozen model, the typical convolution layer contains parameters such as weight tensor, input tensor, forward operation, and output tensor. The quantization operation generally only quantifies the weight or quantifies both the weight and activation. During actual inference, the model still uses floating-point input and output. The operation flow is shown in Figure 4:

Quantifying the trained model will lead to a certain decline in the accuracy of the model (especially for smaller networks). Generally, for TFLite model, the quantization operation is shown in Table 4.

In terms of quantitative comparison of models, TFLite provides the effect comparison of image classification on the Pixel 2 device CPU (where the accuracy refers to the top-1 accuracy), as shown in Table 5:

For the PoseLite model designed in the previous section, the test accuracy comparison of its original model, the TFLite model obtained by direct conversion without quantization, and the TFLite model obtained by weight quantization on the coco2017 dataset is shown in Table 6.

It can be seen that, after the weight quantization of the model, the accuracy of the model decreases to a certain extent. Compared with the original PoseLite model, the mAP decreases by about 8.9 percentage points, while the accuracy of the model directly converted is basically close to that before conversion. The CPU-only test is conducted on Android devices (one plus 7pro). The average delay of the directly converted model (size: 7.8MB) is 567 ms, and the evaluation delay of the weighted model (size: about 2 MB) is 483 ms.

## 3. Results and Analysis

**Figure 5:**
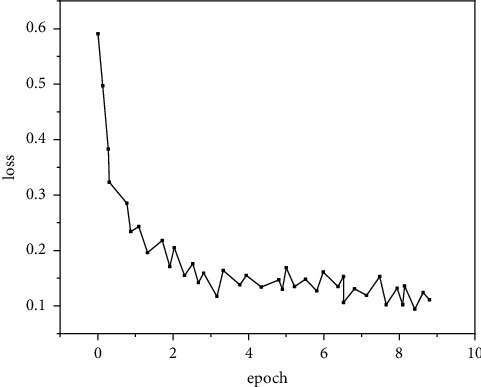
Inspection effect.

**Figure 6:**
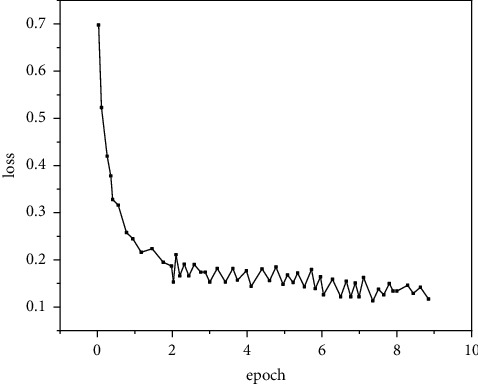
Improved.

In order to verify the detection effect of fast R-CNN for human motion targets after improving the size of the anchor candidate box, the algorithm is experimentally verified in the environment of 64 g memory and NVIDIA 1080ti graphics card. The learning rate is set to 0.001, the number of training iterations is 90000, and the learning rate per 10000 iterations is reduced to 0.1 times of the original. In the training process, a mini batch is used as the training sample input for each iteration; that is, only part of the samples are extracted from the training set for each iteration as the training input. The batch size of this experiment is 128. Experimental comparison is carried out on the INRIA dataset. The changes of the loss value of the model before and after the introduction of the improved anchor candidate box are recorded, respectively, as shown in Figures 5 and 6.

As shown in Figures 5 and 6, the changing trend of loss value before and after improvement is generally rapid at first and then slowly after a certain number of iterations. This is because the model parameters do not have the ability to identify at the beginning, so they go through a process of qualitative change first. At the same time, the phenomenon shows that the loss value will decrease with the increase of the number of iterations, which is due to the use of mini batch in this experiment. Each iteration uses a group of 128 samples from the sample set for forward propagation to calculate the loss function of this round of iteration, resulting in an unstable decline of the loss value. It should be noted that Figure 5 shows the loss value decrease of the native fast R-CNN loss function, while Figure 6 shows the loss value decrease after adjusting the size of the anchor candidate box. It can be clearly found that, after the size of the anchor candidate box is improved, the loss value decreases faster. When the original network iterates to about 20000 times, the loss value decreases nearly gently, while the improved network has leveled off when it iterates to about 10000 times, and the loss value after it becomes stable is also lower (0.15 before the improvement and 0.12 after the improvement). This phenomenon shows that, after a certain number of iterations, the improved network can produce more accurate prediction results, so as to reduce the loss value. Therefore, the experimental results show that the improvement of the anchor candidate box can make the model more suitable for human motion target detection.

**Figure 7:**
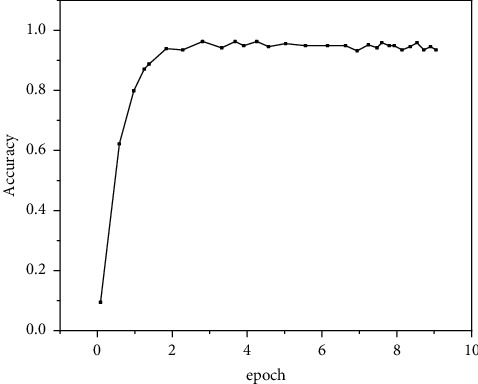
Before improvement.

**Figure 8:**
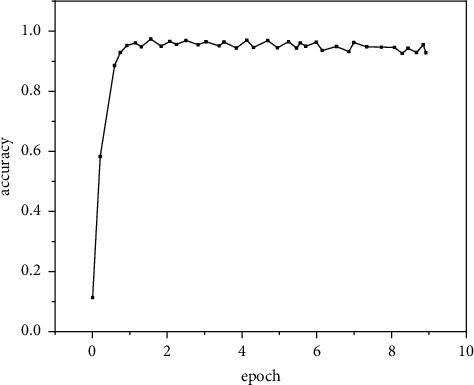
Improved.

The accuracy of the prediction results of the improved algorithm on the INRIA verification set is drawn, as shown in Figures 7 and 8. Experimental results show that the two models basically reach a stable accuracy value after 20000 iterations, and the detection accuracy of the verification set decreases slightly after about 60000 iterations. This is because the model overfits the training parameters of the training set, resulting in the decline of the recognition ability of the model parameters to the verification set [23].

## 4. Conclusion

Human motion recognition is widely used in abnormal behavior detection, athlete action analysis, human-computer interaction, and other fields. Motion recognition based on human joint points is an effective method to improve the accuracy of motion recognition. Therefore, in the field of motion recognition research, the detection of human joint points is a core problem. How to generate accurate joint point positions and how to find the mapping relationship between joint points and motion categories have become a difficult point in this field. Aiming at these two difficulties, an efficient target segmentation network is introduced to generate high-quality joint point prediction masks for the target, and some model improvements are proposed to further improve the detection accuracy of human joint points. Then, designing a fully connected network extracts the mapping relationship between the coordinates of human joint points and motion categories, thus realizing a complete set of action recognition systems based on human keypoint detection. The main research contents and achievements are summarized as follows: the human target detection algorithm based on Faster R-CNN is studied, and the size of the anchor candidate frame in the algorithm is modified for the special model of “person” so that the region of interest generated by the RPN network can have more accurate coverage of character targets. Since a large number of structures of MaskR-CNN are similar to faster R-CNN, this part of the research also lays the foundation for the subsequent research on MaskR-CNN and its improvement methods. The recognition accuracy of the original network before the improvement is 95.2% on the INRIA validation set, and the recognition accuracy after improving the size of the anchor candidate frame is increased to 97.1%, which is 0.19% higher than that before the improvement. It can be seen that the size of the anchor candidate frame has been improved to make the position of the region of interest frame generated during model detection more accurate. After position regression, the output target frame can be closer to the real annotation frame in size and position, thereby improving the recognition accuracy. This high-precision motion recognition technology can be applied to competitions, human motion training, physical exercise, fitness guidance, and other motion recognition scenes. Due to the rich variety of character actions, the high similarity of a large number of actions, and the rich action scenes of characters, there are still many valuable research directions in the process of character motion recognition based on joint point detection such as human-computer interaction and athlete motion analysis.

## Figures and Tables

**Table 1:** Size of improved anchor candidate box.

Candidate box number	Candidate box size (width ∗ height unit: pixel)
Candidate box 1	20 × 50
Candidate box 2	24 × 60
Candidate box 3	32 × 80
Candidate box 4	44 × 11
Candidate box 5	60 × 150
Candidate box 6	80 × 200
Candidate box 7	120 × 300
Candidate box 8	160 × 400
Candidate box 9	200 × 500

**Table 2:** Anchor candidate box size based on the K-means clustering algorithm.

Candidate box number	Candidate box size (width ∗ height unit: pixel)
Candidate box 1	18 × 44
Candidate box 2	22 × 55
Candidate box 3	31 × 76
Candidate box 4	43 × 105
Candidate box 5	59 × 145
Candidate box 6	82 × 200
Candidate box 7	113 × 276
Candidate box 8	156 × 381
Candidate box 9	215 × 526
Candidate box 10	45 × 20
Candidate box 11	110 × 42
Candidate box 12	250 × 100
Candidate box 13	500 × 204

**Table 3:** Several bit bits and their data range.

Digit (bit)	Minimum value	Maximum
8	−120	120
16	−32766	32766
32	−2147483640	2147483640

**Table 4:** Several quantization operation modes are supported by TFLite.

Quantitative method	Advantage	Applicable hardware
Weight quantization	The model is reduced by 4 times and accelerated by 2-3 times, with good accuracy	CPU
All integer quantization	The model is reduced by 4 times and accelerated by more than 3 times	CPU, edge TPU, etc.
Float 16 quantization	The model is reduced by 2 times, which has the potential of GPU acceleration	CPU/GPU

**Table 5:** Comparison of quantitative effects of various models.

Model	Precision: raw	Precision quantization	Delay: initial	Delay quantization (ms)	Size: initial (MB)	Size quantization (MB)
MobileNetV1	0.708	0.654	124 ms	112	16.6	4.3
MobileNetV2	0.709	0.632	89 ms^3^	98	13	3.6
InceptionV3	0.77	0.762	1130 ms	845	95.9	23.9
ResnetV2	0.76	0.758	973 ms	2868	178.5	44.9

**Table 6:** Comparison of test accuracy of three models on coco2017 dataset.

Model	PoseLite	Direct conversion model	Weight quantization model
Test data set	2017 val	2017 val	2017 val
AP@0.5 : 0.95	36.9	35.9	27.6
AP@0.5	64.0	62.8	53.8
AP@0.75	35.0	33.4	26.1
AP medium	30.1	28.1	28.2
AP large	46.0	44.0	30.8
AR@0.5 : 0.95	41.5	40.9	32.6
AR@0.5	66.1	64.7	56.9
AR@0.75	40.7	38.7	29.0
AR medium	31.5	29.9	28.9
AR large	55.6	53.4	35.7

## Data Availability

The raw data used to support the findings of the study can be obtained from the corresponding author upon request.
